# Xenobiotics, Trace Metals and Genetics in the Pathogenesis of Tauopathies

**DOI:** 10.3390/ijerph17041269

**Published:** 2020-02-17

**Authors:** Jan Aaseth, Aleksandra Buha, David R. Wallace, Geir Bjørklund

**Affiliations:** 1 Research Department, Innlandet Hospital Trust, 2380 Brumunddal, Norway; 2 Department of Toxicology “Akademik Danilo Soldatović”, Faculty of Pharmacy, University of Belgrade, 11000 Belgrade, Serbia; aleksandra.buha@pharmacy.bg.ac.rs; 3 Center for Health Sciences, School of Biomedical Sciences, Oklahoma State University, Tulsa, OK 74107, USA; 4 Council for Nutritional and Environmental Medicine (CONEM), 8610 Mo i Rana, Norway; bjorklund@conem.org

**Keywords:** xenobiotics, metals, pathogenesis, tauopathies

## Abstract

Tauopathies are a disease group characterized by either pathological accumulation or release of fragments of hyperphosphorylated tau proteins originating from the central nervous system. The tau hypotheses of Parkinson’s and Alzheimer’s diseases contain a clinically diverse spectrum of tauopathies. Studies of case records of various tauopathies may reveal clinical phenotype characteristics of the disease. In addition, improved understanding of different tauopathies would disclose environmental factors, such as xenobiotics and trace metals, that can precipitate or modify the progression of the disorder. Important for diagnostics and monitoring of these disorders is a further development of adequate biomarkers, including refined neuroimaging, or proteomics. Our goal is to provide an in-depth review of the current literature regarding the pathophysiological roles of tau proteins and the pathogenic factors leading to various tauopathies, with the perspective of future advances in potential therapeutic strategies.

## 1. Introduction

In medical terminology, tauopathies generally refer to neurodegenerative disorders with pathological tau protein accumulation in the central nervous system, especially in neurons. Physiologically, tau is a microtubule-associated protein expressed in neurons. Abnormal fibrillary tangles are formed by soluble tau proteins accumulated within a cell. Hyperphosphorylated derivatives of soluble tau proteins can detach from microtubules. Improved understanding of the composition of pathological tau aggregates will aid clinicians in the future by providing more information, which will result in improved etiological and clinical diagnosis of overlapping diseases. Some tauopathies leading to Parkinsonism disorders have been shown to at least partially respond to levodopa therapy. In contrast, other tauopathies are distinguished by a rapidly or slowly developing dementia, and may be diagnosed as a frontal lobe impairment or Alzheimer’s disease. Other tauopathies are motor neuron disorders, which are clinically recognized as amyotrophic lateral sclerosis. An increased number of tauopathies has been diagnosed in various geographic regions (New Guinea, Guadeloupe, Guam, etc.). The vast geographic distribution of different tauopathies represents a challenge when determining the etiological environmental factors of these diseases. The current state of research in the area of tauopathies supports the significant need for future research to consider environmental etiologies of post-traumatic and post-apoplectic tauopathies, as well as post-encephalitic symptoms of Parkinsonism.

Here, we review the current knowledge concerning tau proteins, tauopathies, and tau that is presumed to be a pathogenic factor in several neurodegenerative diseases, with the perspective that increasing insight will lead to new approaches in management and prevention.

## 2. Functional Roles of Tau Proteins

Under physiological conditions, tau proteins are highly soluble microtubule-associated proteins “tau” (MAPT). In humans, tau proteins are mainly found in neurons and also, although to a lesser extent, in non-neuronal cells [1]. The primary function of tau is to ensure that axonal microtubules are stable. The activity of tau is primarily in the axons’ distal part, whereas other microtubule-associated proteins (MAPs) operate in dendrites and the proximal portions. Additional functions of tau proteins include the regulation of microtubule-mediated transport of nutrients [2].

In humans, tau proteins are encoded by the *MAPT* gene, which consists of 16 exons positioned on chromosome 17q21 [3]. In human adult brains, six tau protein isoforms (types) ranging from 352 to 441 amino acids are produced. In the two haplogroups (H1 and H2) of the *MAPT* gene, the gene is presented inverted. Haplogroup H2 is more common in Europe, although haplogroup H1 is also found frequently. Haplogroup H1 seems associated with an elevated probability of certain dementias, such as Alzheimer’s disease. Since both haplogroups are present in Europe, the recombination between the inverted haplotypes may possibly cause one functional copy of the gene to be missing, leading to congenital disabilities such as esophageal atresia and congenital heart defect [4].

Tau has 79 possible sites for phosphorylation on multiple serine (Ser) and threonine (Thr) residues on the most extended tau isoform. Kinases regulate phosphorylation of tau, for instance, the serine/threonine kinase (PKN). Activation of the PKN causes rapid phosphorylation of tau, which disrupts microtubule organization [5]. Physiologically, the degree of tau phosphorylation, regardless of the isoform, declines with age because of increased activity of phosphatases [6]. The phosphatases play a critical role due to their ability to dephosphorylate phospho-tau.

Pathological aggregation due to hyperphosphorylation of tau in neurons causes neurofibrillary cellular degeneration. The mechanism behind the propagation of pathological MAPTs from cell to cell is not yet identified. However, several mechanisms of propagation have been suggested, including synaptic and also non-synaptic transfer mechanisms [7]. Among the factors that appear to favor pathological fibrillation and propagation are excessive hyperphosphorylation, together with increased local levels of zinc ions, which may displace copper from essential locations [8]. These observations support the presumption that not only genetic defects, but also post-translational impacts due to environmental factors can promote development of a tauopathy.

Hyperphosphorylation of tau proteins can cause aggregation of tangles that consist of straight and paired helical filaments, which appear to play an etiological role in different tauopathies, including frontotemporal dementia and Alzheimer’s disease [9]. Upon misfolding, tau shifts from a soluble protein under normal physiological conditions to a very insoluble protein. The formation of insoluble proteins is accompanied by disruption of the cytoskeleton and protein aggregation that contributes to several neurodegenerative diseases. Due to the formation of apparently toxic tangles [10], insoluble tau proteins directly affect the breakdown of living cells, which then interrupts nerve synapse activity. Neurofibrillary tangles are aggregates of tau proteins that block the transport/distribution of essential nutrients throughout brain cells, and ultimately result in cell deterioration and death [11].

## 3. Clinical Types of Tauopathies

### 3.1. The Tau Hypothesis of Alzheimer’s Disease

**Figure 1:**
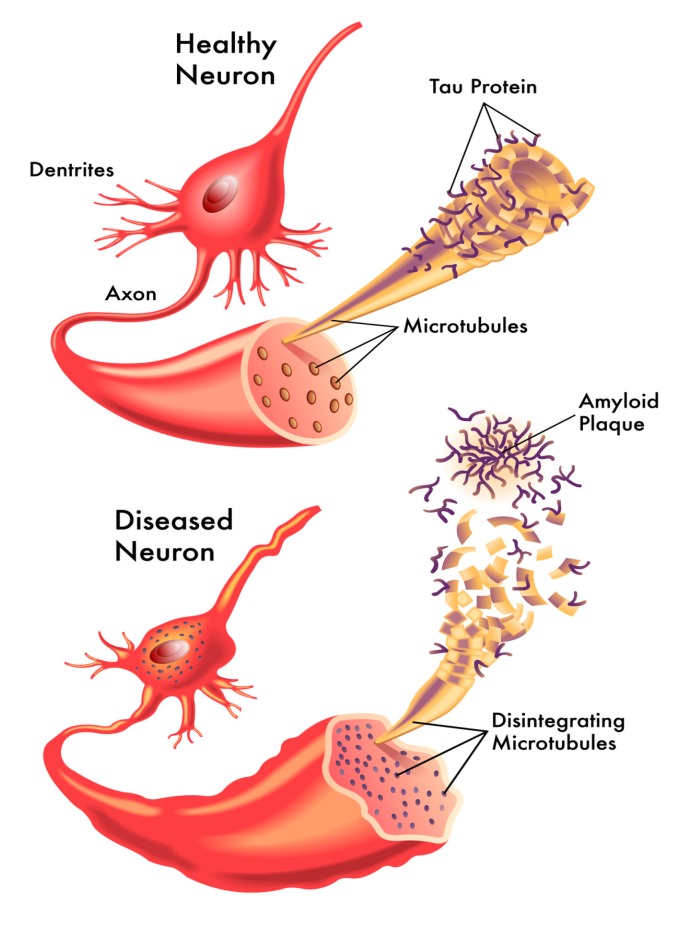
Schematic representation of a healthy (**top**) and a diseased (**bottom**) neuron in a normal brain and a brain with Alzheimer’s disease (AD). In the healthy neuron, you can see the organized structure of the microtubules, and the involvement of the tau protein. In the diseased neuron, the microtubules are disintegrating, and there is a loss of organization and structure of the tau proteins accompanied by plaque formation and the eventual degeneration of the neuron.

Most cases of Alzheimer’s disease (AD) are sporadic, and environmental factors may play an important pathogenetic role in them (Figure 1). The tau hypothesis of AD states that abnormal or excessive tau phosphorylation is a crucial early event in AD development, resulting in neurofibrillary tangles (NFTs) [12]. In AD, several tau amino acids are phosphorylated, and pre-NFT phosphorylation occurs at serine 119, 202, 409, and at combinations of the three serine sites. In AD, all the six isoforms of tau may occur in a hyperphosphorylated state of paired helical filaments.

The exact causes of initiation and acceleration of tau accumulation in the absence of mutations are not yet known, but are thought to result from unregulated phosphorylation, which may be induced by environmental toxins. In AD, increased activity of cyclin-dependent-kinase 5 (CDK5) has been reported, and this kinase is associated with neurofibrillary tangles and elevated intracellular calcium [13,14]. Since selenium compounds can reduce the phosphorylation of tau in cell cultures as well as in mouse models of AD, it is thought that oxidative stress may be a kinase activator [15]. According to the tau hypothesis of AD, the primary characteristic is the formation of NFTs consisting of the hyperphosphorylated tau protein. The amyloid hypothesis of AD is characterized by extracellular plaque formation with an insoluble amyloid (A) β-peptide fragment-42, which consists of the amyloid core. Comparing the amyloid hypothesis with the tau hypothesis, the tau hypothesis suggests that intracellular tangles are the first to form, followed by the extracellular plaques. Current evidence correlates the incidences of severe cognitive deficits with the presence of NFTs, and not amyloids [16,17]. 

### 3.2. Parkinson’s Disease

Genetic mutations linked to familial Parkinson’s disease (PD), such as in the Parkin gene and the gene for LRRK2 (leucine-rich repeat kinase 2), are associated with mitochondrial dysfunction [18,19]. In contrast, the MAPT gene seems not to be involved directly in the etiology of classical PD [20]. The association of familial PD with mitochondrial dysfunction links changes in mitochondrial function to PD pathogenesis. Although some of the factors that genetically increase the risk of PD development have been identified, most PD cases are sporadic and dependent upon environmental factors [21]. The essential role of environmental exposure has been strengthened by research on Parkinsonism due to MPTP (1-methyl-4-phenyl-1,2,3,6-tetrahydropyridine) [22]. Exposure to environmental/mitochondrial toxins, including MPTP, rotenone, and paraquat, increases the risk of getting PD [23]. These toxins are inhibitors of mitochondrial complex I, and cause neuronal death in substantia nigra and Parkinsonism in experimental models. Inhibition of mitochondrial complex I and/or elevated mitochondrial iron in susceptible loci cause oxidative stress; that explains the observations of reduced glutathione levels in these cells [24] and may, as a result, disturb the post-translational balance between phosphorylation and dephosphorylation of the tau protein. Other environmental toxicants, including manganese, have been suspected of increasing the risk of oxidative stress and PD development [25,26]. In synaptically enriched parts of the frontotemporal cortex, phosphorylated tau (Table 1) is considered a characteristic trait of advanced PD [27]. Tau hyperphosphorylation has been observed to occur parallel to the presence of α-synuclein aggregation [28], although in advanced PD, intracellular insoluble aggregates of α-synuclein (Lewy bodies) are the hallmarks. Progression of PD appears to depend on the toxicity of environmental pollutants. The pollutants include pesticides and metal ions (including iron, manganese, aluminum, and cadmium) which appear to accelerate the aggregation of α-synuclein and tau [26,29,30].

### 3.3. Progressive Supranuclear Palsy

Tau pathology is thought to be predominant in progressive supranuclear palsy (PSP), which is clinically a type of atypical Parkinsonism. Clinically, the features are reasonably well defined [31]. However, the early signs are unspecific, which may delay the specific diagnosis, often up to at least four years after the initial signs of disease. Early symptoms and signs are usually moderately impaired mobility and slowly developing cognitive deficits. PSP variants have been described, such as a Parkinson-mimicking form that is responsive to levodopa, and also a more akinetic form dominated by gait and speech problems [32]. Recently, in northern France, a PSP clustering was reported in a district around Wattrelos. This district was previously known for its textile dyeing and tanneries during the 20th century, both processes utilizing chromate and arsenic obtained from the chemical plants nearby. It was found that the soil in these regions was contaminated with hexavalent chromium [33]. It is known that chromate, which rapidly traverses biological membranes [34], exerts toxic effects on mitochondria [35,36]. Another cluster with PSP-resembling symptomatology has been described in a region that was previously active in iron and copper mining in eastern Norway [37]. This tauopathy was associated with the well-known mitochondrial toxicity of non-complexed iron [38]. The neuropathology of PSP is distinguished by midbrain atrophy and involvement of pallidum and thalamus, but only modest frontal engagement.

### 3.4. Examples of Geographic Clusters of Tauopathies

In distinct geographical areas, tauopathies have been found to include amyotrophic lateral sclerosis/Parkinson–dementia complex (ALS/PDC) of New Guinea, Guam, and the Kii peninsula [39]. Each of the distinct tauopathies exhibits case symptomology and neuropathological changes similar to PSP. For several decades, the ALS/PDC had been occurring in native Chamorro families in these areas, but today the incidence has been substantially reduced. Still, the primary causative factors are unknown. One hypothesis is that exposure to the non-proteinogenic amino acid β-methylamino-l-alanine (BMAA) leads to the development of Guam disease. Yet, verification of this mechanism has not been successful. It has been hypothesized that persons with atypical Parkinsonism living in the French West Indies have developed the condition due to the ingestion of a tropical plant [40], which contains mitochondrial toxins, such as isoquinolines and annonacin. In animal models, exposure to the same compounds has been shown to cause neurodegeneration [41].

Further research into possible mitochondrial toxins in the environment of the Chamorro population in Guam would be of interest. On Martinique and Guadeloupe islands, the disease-associated pathology of several patients resembles PSP, although these cases are considered unclassified non-dopa-responsive Parkinsonism. Reports of two postmortem autopsy cases suggest the disorder was due to a synucleinopathy with Lewy bodies in the substantia nigra [42].

### 3.5. Frontotemporal Dementia and Pick’s Disease

In frontotemporal dementia, different mutations in the MAPT gene associated with chromosome 17 cause various clinical presentations [43]. The age at onset can be as low as 20 years, and the disease may extend well into later life. The deterioration during the disease goes on for at least ten years. Clinically, the frontal variant of AD is the dominant phenotype. The parkinsonian component may resemble a classical, although not a dopa-responsive syndrome. Although a motor neuron component is not considered a usual symptom in cases with MAPT mutations, the K317M mutation is associated with amyotrophy characterized by fasciculations and motoric denervation. Pick’s disease may be considered a subtype characterized by circumscribed atrophy of the fronto-parieto-temporal cortex, with neurons containing Pick bodies, i.e., neuronal accumulation of hyperphosphorylated tau. While other pathologies causing frontotemporal lobar degeneration are associated with a genetic cause, the evidence is not conclusive on whether Pick’s disease has a direct genetic cause. However, it is known to be a hereditary disease, and mutations in the tau gene have been associated with some cases [44]. Nonetheless, environmental factors appear to modify the symptoms [45].

## 4. Genetic Factors

Environmental factors, including mitochondrial toxins, appear to play a significant role in the pathogenesis of the various tauopathies, although genetic alterations have been identified in several of the disease entities. Although approximately 90% of Alzheimer’s disease cases occur sporadically, mutated genes coding for either amyloid precursor protein (chromosome 21) or presenilin-1 (PS1; chromosome 14) or presenilin-2 (PS2; chromosome 1) are found in early-onset familial forms of AD [46]. Furthermore, many sporadic AD patients are carriers of the e4 allele of the ApoE gene (apolipoprotein E; chromosome 19) [47]. In addition, today, the genome-wide association studies of AD are nearing identification of a wide range of genetic loci influencing the risk of AD [48]. Regarding other tauopathies, the cases with the mutated tau gene (FTDP-17) [38] make up a substantial fraction of those with corticobasal degeneration or frontotemporal dementia [26]. However, the majority of patients with Parkinson’s disease, PSP, or Pick’s disease are regarded in the literature as sporadic [35]. Nevertheless, it is known that mutations in the tau gene may affect microtubule construction, resulting in increased tau self-aggregation. A recent review by Strang et al. [49] discusses the impact of MAPT mutations on tauopathies and neurodegeneration. Since the initial discovery in 1998 [50], several families with MAPT mutations and progressive phenotypes similar to PSP have been identified [51]. PARK2 mutations also appear to cause a PSP-like picture. It is also known that LRRK2 gene mutations can cause familial, autosomal-dominant Parkinsonism. These familial or autosomal-dominant cases may present as PSP-like Parkinsonism, yet rarely cause PSP-like conditions.

At present, tau haplotype MAPT H1 is the only genetically consistent confirmed risk locus of PSP [52]. However, genetic research has also identified a risk locus for PSP on chromosome 11p11-12 [53]. A family from Extremadura in Spain was found to have a locus on chromosome 1q31 [54], but the specific mutation has not been identified [32]. More research on the risk loci and their associations with PSP is required.

## 5. Therapeutic and Preventive Possibilities

Although direct corrections of MAPT mutations are not yet possible, defective post-translational handling of the tau protein can be modified or prevented. Avoiding exposure to hazardous metals and metal mixtures, as well as to agents with potentially toxic effects on mitochondria is considered important for the prevention of mutations and tauopathies. In developing the tau pathology, mitochondrial dysfunction has been thought to be critical. For sporadic PSP, Guadeloupe disease can be used as a possible model. As discussed above, the intake of Annonaceae plants is a risk factor for developing the disease and mitochondrial toxins, like isoquinoline alkaloids, are hypothesized as the toxic principle. Annonaceae plants have been proven toxic in vitro due to potent complex I inhibition [55,56]. After experimental administration, these compounds enter a rodent’s brain and selectively destroy nigral and striatal neurons [57]. Beyond the fact that mitochondrial dysfunction plays a role in PSP [58], pilot trials, either with coenzyme Q10 or with a pyruvate/niacinamide combination, have been proposed [59].

Regarding therapeutic possibilities, increased activity of both glycogen synthase kinase-3 (GSK-3) and GSK-5 result in the hyperphosphorylation of tau at relevant sites [60]. Transgenic mice overexpressing wild-type GSK-3 were obtained to investigate if GSK-3 inhibition has therapeutic potential. The genetically modified mice showed histopathological changes (neuronal loss and elevated tau phosphorylation) and reduced spatial recognition [61]. The histological changes and reduced spatial recognition in GSK-3-overexpressing mice supports the hypothesis that GSK-3 inhibitors have therapeutic potential. Furthermore, treatment using lithium functioning as a GSK-3 inhibitor has been reported to prevent neuropathological alterations in mice with elevated GSK-3 activity [62].

Chronic inflammation plays a crucial pathogenetic role in tauopathies, as inflammation can promote the development of various tauopathies. Neuroinflammation accompanied by vascular and/or synaptic dysfunction may be the triggering cause of disease in AD, as well as in other tauopathies [63]. Elevated TNFα has been reported, and treatment with anti-TNF-agents has been proposed as a potential therapy [64]. Similar to neuroinflammation in AD and the potential use of anti-TNF agents, the neuroinflammation in PD is widespread, and TNFα is elevated in both serum and cerebrospinal fluid [65]. Retrospective studies [66] and meta-analyses [67] indicate that NSAIDs have disease-modifying potential due to their ability to attenuate the neuroinflammation that can be a precursor to tauopathy development.

Several other compounds can inhibit the accumulation of tau, including anthraquinones (emodin, daunorubicin, and adriamycin) and polyphenols [68]. Polyphenols may be “simple” or “complex”. Simple polyphenols are phenolic acid derivatives, such as rosmarinic acid, and complex polyphenols can be flavonoids (catechin), tannins (tannic acid), and others, such as stilbenes and resveratrol [69]. Such compounds appear in cell models to relieve the toxicity due to tau aggregation. A significant level of caution must be used when interpreting the in vitro results for polyphenols and flavonoids. Poly-ringed structures, such as polyphenols, flavonoids, and others, can fall into a category referred to as the “pan-assay interference compounds”, or “PAINS.” The PAINS are characterized by having multiple mechanisms of action, and are known to yield false positives in screening assays due to their ability to act through multiple nonspecific pathways. Jonathan Beall’s laboratory has been at the forefront in the research on the PAINS and how to control for the false positives elicited by the PAINS in high-throughput screening [70,71,72]. Researchers need to utilize caution when interpreting results with the compounds that may fall into the PAIN category. In an attempt to discern actual versus artifact response, Baell and Nissink [70] attempted to develop a “filtering” system to block the influence of PAIN compounds. The future of compounds which may be considered PAINS should be viewed with cautious optimism, with the understanding that the mechanisms of their effect may be much more complex than simple bimolecular interactions. Recent studies on animal models have confirmed the ability of anthraquinones and polyphenols to either prevent the accumulation of tau, or “dissolve” the existing tau aggregates [68,69,73]. Transgenic mice were first developed in the late 1990s and were instrumental in advancing our understanding of tauopathies and critical for the development of potential therapies [74]. In the last two decades, there have only been small strides forward in the development of additional model systems or therapies [75,76].

In addition to the development of better animal models, clinical trials are in need of more accurate clinical assessments of PSP and other tauopathies. As better assessments are developed with improved accuracy, the focus must be maintained on the end-points of therapeutic intervention, i.e., the Unified Parkinson’s Disease Rating Scale (UPDRS) in addition to relevant cognitive examination scales.

## 6. Conclusions

The present review demonstrates that symptomatic monitoring and early diagnosis of tauopathies, including sporadic Alzheimer’s disease and classical or atypical Parkinsonism, are possible. To achieve symptomatic monitoring and early diagnosis, the development of future trials and clinical follow-ups is clinically important. Results obtained using mouse models, such as those with transgenic mice, can be utilized for designing trials of novel drugs for clinical therapeutic developments. However, the extrapolation of murine data to human therapy has proven difficult. Complicating factors may overlap the etiologies of tauopathies together with the complicated interplay between genetics and the environment (e.g., Guam disease).

Valid and reliable biomarkers for translational research are required and must be further improved. At present, a possible quantifiable therapeutic target is the neuroinflammation that accompanies tauopathies, presumably used together with cognitive scores. Interestingly, several well-known drugs, including NSAIDs and lithium, can target crucial pathogenic steps. To identify strategies to modify the development of tauopathies is of imperative importance, although the development of clinically potent principles may be time consuming.

## Figures and Tables

**Table 1:** Different types of phosphorylated tauopathies, their location, and their management.

Disease	Location	Management
Alzheimer’s disease	Hippocampus and entorhinal cortex	Cholinesterase inhibitors, memantine
Parkinson’s disease	Substantia nigra in the basal ganglia	Levodopa
Progressive supranuclear palsy	Basal ganglia and brain stem/spinal cord	Levodopa (in some cases only)
Frontotemporal dementia	Frontal and temporal lobes	Antipsychotics and antidepressants
Chronic traumatic encephalopathy	Sulcus depths	No approved therapy
